# Incidence and survival of end-stage kidney disease due to polycystic kidney disease in Australia and New Zealand (1963–2014)

**DOI:** 10.1186/s12963-017-0123-7

**Published:** 2017-02-17

**Authors:** Mangalee R. Fernando, Hannah Dent, Stephen P. McDonald, Gopala K. Rangan

**Affiliations:** 1 Department of Renal Medicine, Westmead Hospital, Western Sydney Local Health District, Sydney, Australia; 2 Department of Nephrology, Prince of Wales Hospital, Randwick, Sydney, Australia; 3 Prince of Wales Clinical School, University of New South Wales, Sydney, Australia; 4 ANZDATA Registry, Adelaide, Australia; 5 Department of Medicine, The University of Adelaide, Adelaide, Australia; 6 Centre for Transplant and Renal Research, Westmead Institute for Medical Research, The University of Sydney, PO Box 412, 176 Hawkesbury Road, Westmead, Sydney, NSW 2145 Australia

**Keywords:** End-stage kidney disease, Polycystic kidney disease

## Abstract

**Background:**

The aim of this study was to determine whether the incidence and survival of patients with end-stage kidney disease (ESKD) due to polycystic kidney disease (PKD) has changed in Australia and New Zealand.

**Methods:**

Data for all PKD patients who developed ESKD and commenced renal replacement therapy (RRT) was assessed using the Australia and New Zealand Dialysis and Transplant Registry from 1963 to 2014.

**Results:**

A total 4678 patients with ESKD due to PKD received RRT during the study period. The incidence rate of ESKD (per million population per year) due to PKD rose by 3.2-fold (1970–2010), but the percentage increase between each decade decreased (54.4, 43.8, 25.6 and 6.57%). The median age of onset of new patients developing ESKD has been stable since 1990. Haemodialysis was the most common initial mode of RRT (between 62 and 76% of patients) whereas 24–29% received peritoneal dialysis. The 5-year survival rate of PKD patients on RRT (censored for transplantation and adjusted for age) improved from 26 to 84%, with the percentage increase between each successive time period being 123, 7, 21, 19 and 7.4%. The percentage of deaths on RRT due to cerebrovascular disease declined from 15 to 6%.

**Conclusions:**

The incidence and age of onset of ESKD due to PKD has remained unchanged in the modern era though patient survival on RRT has continued to improve. These data suggest that the development and implementation of disease-specific treatments prior to RRT is needed to effectively diminish the incidence of ESKD due to PKD.

## Background

Autosomal dominant polycystic kidney disease (ADPKD) is the most common monogenetic disorder causing end-stage kidney disease (ESKD) in adults [1–5]. It is a slowly progressive adult-onset chronic kidney disease that is characterized by the formation and growth of numerous fluid-filled cysts in the kidneys, which cause renal enlargement and pain, hypertension, intracranial aneurysms and cardiovascular disease [4]. ADPKD is caused by heterozygous germ-line mutations in *PKD1* (85% of cases) and to a lesser extent *PKD2* (15% of cases) [6]. These genes encode the proteins polycystin-1 and polycystin-2, which maintain the normal geometric structure of the distal nephron in the kidney [7]. Patients with *PKD2* and hypomorphic *PKD1* mutations have a better renal prognosis and a delayed onset of ESKD, compared to patients with truncating *PKD1* mutations [7].

Previous observational studies, predominantly of dialysis registry data, have shown that the age of onset of ESKD due to ADPKD has increased over time and that patient survival on renal replacement therapy (RRT) has also improved [3, 8–11]. Orskov et al. reported that the survival of Danish patients with ESKD treated with RRT has steadily increased over an 18-year period from 1990 to 2007 [11]. Similarly, a single-center study from the Oxford Renal Unit showed that patient survival improved over a 40-year period (1971–2000). Lastly, dialysis registry data from Europe (1991–2010) showed that the incidence rate of ESKD due to ADPKD has increased slightly over the last two decades (1991–2009) [12].

Over the last 50 years, there have been major improvements in the management of hypertension, hyperlipidemia and the treatment of anemia due to ESKD [13]. In Australia, angiotensin-converting enzyme inhibitors were first introduced onto the national Pharmaceutical Benefits Scheme (a government subsidized program for prescription drugs) in 1983, whereas erythropoietin stimulating agents and angiotensin receptor blockers became available in 1992 and 1997 respectively. Lowering blood pressure and treatment with cholesterol-lowering agents reduces the rate of renal cyst growth in ADPKD and could potentially delay the age of onset of ESKD [2, 14, 15]. Moreover, the treatment of anemia associated with ESKD improves outcomes in patients receiving RRT [16]. The Australia and New Zealand Dialysis and Transplant Registry (ANZDATA) has been recording the incidence, prevalence and outcome of all maintenance dialysis and transplant patients in Australia and New Zealand since 1963 [17]. This registry therefore provides an extensive timeframe to determine whether improvements in medical therapy might precede changes in the incidence of ESKD due to PKD and patient survival on RRT. Thus, the aim of the study was to determine whether there have been time-specific changes in the incidence of ESKD due to PKD and survival rates on RRT in Australia and New Zealand.

## Methods

### Patient population

The ANZDATA registry records the incidence, prevalence and outcome of all maintenance dialysis and transplant patients in Australia and New Zealand. Data are collected by means of survey forms for each patient at 6-month intervals until 2004, after which it was altered to a yearly survey ending on December 31, together with notification of key events in real time The cohort study included all patients with ESKD with or without PKD enrolled in the ANZDATA commencing RRT in Australia and New Zealand between May 15, 1963 and December 31, 2014. Patients who commenced RRT overseas were excluded.

All patients entered into the ANZDATA were considered by the treating nephrologist to have ESKD due to PKD and therefore thought to require long-term RRT at the time they were enrolled in the registry. The assignment of the primary renal disease as “polycystic kidney disease” is made by the treating nephrologist on clinical grounds. This definition has been unchanged over the course of the study. There is no requirement for confirmatory genetic testing. Treatment modality (dialysis vs. pre-emptive transplant) was assigned at the commencement of RRT. The primary outcomes examined were the incidence rate and age of onset of ESKD as well as the survival rate and characteristics of RRT. The treatment era was determined by the date of commencing dialysis, and divided into 10-year periods: 1963–1974, 1975–1984, 1985–1994, 1995–1994, 2005–2014.

### Statistical analysis

Analyses were performed on a de-identified extract from the ANZDATA Registry. Confidence intervals for incidence rates were calculated using a Poisson distribution, which were also used to compare rates between groups. Patient survival and renal allograft survival was estimated by Kaplan–Meier analysis; age-adjusted curves were adjusted to median age at dialysis start. Patients were not censored in the survival analysis when the dialysis modality was switched from peritoneal dialysis to hemodialysis or vice versa. Differences between Kaplan-Meier curves were tested using the log-rank test. Competing risk analyses of cumulative incidence were used for the incidence of transplantation (with death as a competing risk). The non-PKD group is shown as “Total” or sub-divided into “Vascular/Diabetes” and “Non-vascular” causes. Data were analysed using Stata/IC 14.1 (Stata Corporation, College Station, TX, USA). *P*-values <0.05 were considered statistically significant.

## Results

### Incidence rate and median age of onset of renal replacement therapy in PKD

A total 4678 patients with ESKD due to PKD received RRT or underwent pre-emptive renal transplantation in Australia and New Zealand between 1963 and 2014, from a population of 70,544 (or 6.6% of total). Of the 4678 patients, 23 were of Aboriginal/Torres Strait Islander heritage (total population of 4534) and 98 were of Maori/Islander descent (total population of 6661).

The incident rate of ESKD due to PKD (as expressed as per million population) increased by 3.2-fold between 1970 and 2010 (Table 1). However, the percentage increase in the incidence rates between each successive decade decreased between 1970 and 2010 (22.5; 56.8, 43.3 and 35.8%).

**Table 1:** Incidence of end-stage kidney disease to PKD from 1970 to 2010 (incidence rates expressed per million population per year, 95% confidence interval [CI])

Year	Incident Rate of ESKD due to PKD (million population per year)	Lower 95% CI	Upper 95% CI
1970–1974	2.04	1.71	2.42
1975–1979	2.50	2.18	2.85
1980–1984	3.15^*^	2.80	3.54
1985–1989	3.92^*^	3.54	4.33
1990–1994	4.53^*^	4.13	4.95
1995–1999	5.62^*^	5.18	6.08
2000–2004	5.69^*^	5.26	6.14
2005–2009	7.63^*^	7.15	8.12
2010–2014	6.57^*^	6.15	7.02

^*^ *P* < 0.001 compared to 1970

**Fig. 1:**
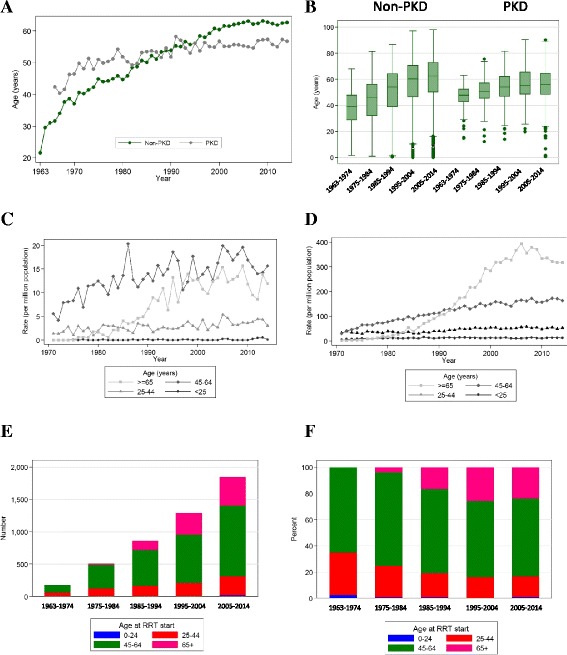
Changes in the age of onset of end-stage kidney disease due to PKD. **a** Median age of onset over the duration of the study (1963–2014); **b** Median age of onset according to the time period; for both PKD and non-PKD groups the age of incidence varies significantly over time (*P* < 0.001); **c** Incidence of ESKD due to PKD according to age group; **d** Incidence of ESKD due to non-PKD according to age group; **e** Absolute numbers of PKD patients, according to age group, receiving RRT over the study period; **f** Percentage of PKD patients according to age, over the study period

The median age of onset of ESKD due to PKD increased over time but has been relatively stable since 1990 (Fig. 1a and b). Prior to 1990, the median age of onset of ESKD in PKD patients was higher than in patients without PKD (i.e., the non-PKD group), whereas in the most recent time-period (1990–2014) this has been reversed (Fig. 1b).

The changes in the incidence of RRT and absolute numbers of patients, according to age category, are shown in Fig. 1c–f. Overall, the incidence of ESKD in PKD patients younger than 25 years of age has been a relatively small proportion of the total RRT population, and has been stable over time (Fig. 1c). In the other categories, the incidence of ESKD due to PKD in patients aged between 25 and 44 years and ≥65, increased over time but has been relatively stable since 1990 (Fig. 1c, e and f). In contrast in the non-PKD population, the number of new patients ≥65 years of age who commenced RRT has rapidly increased since 1990 (Fig. 1c and d). The changes in the absolute numbers and the age-specific proportions are shown in Fig. 1e and f. Patients aged between 45 and 64 years of age were the most common category in all time periods, representing between approximately half to three-quarters of the PKD population on RRT. Since 1980, there has been an increase in the proportion of patients older than 65 years of age (Fig. 1f).

### Characteristics of the initial modality of renal replacement therapy in PKD

**Fig. 2:**
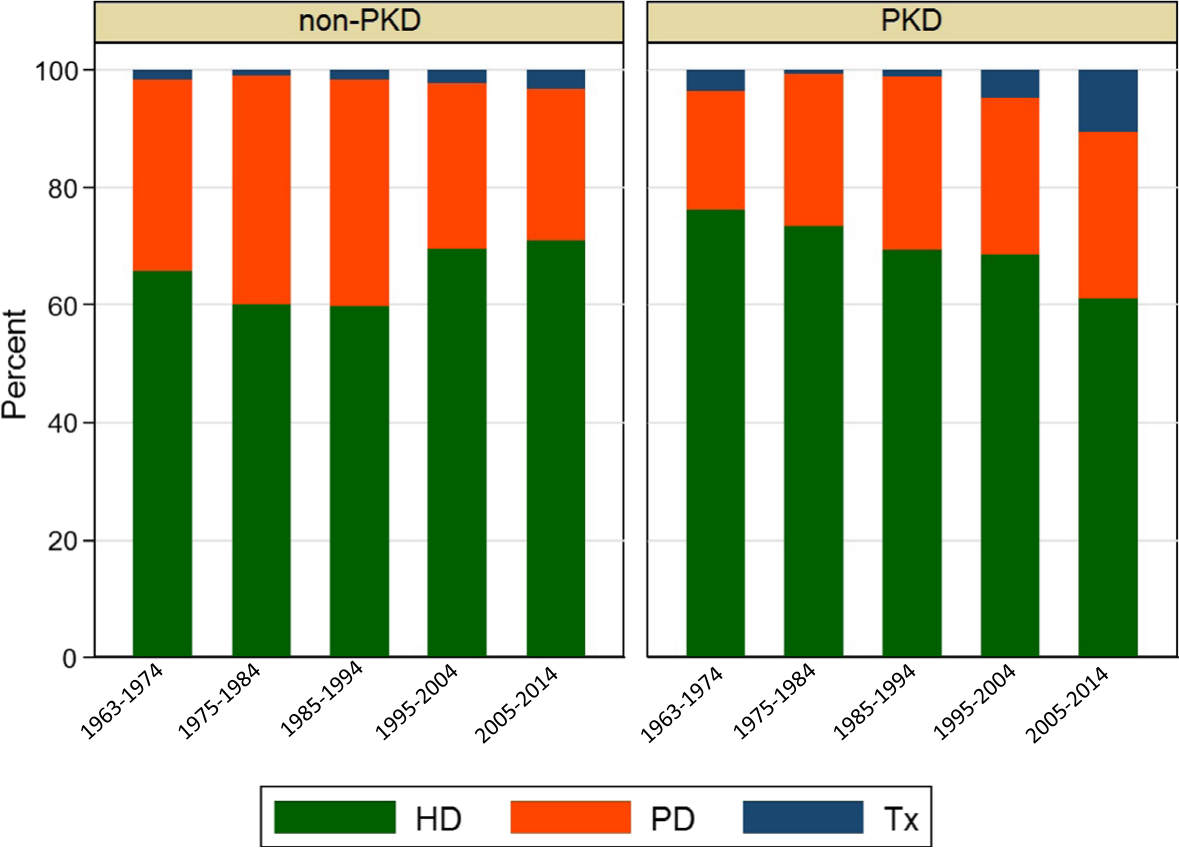
Changes in the relative proportions initial RRT modality in non-PKD and PKD patients according to the time-period. For each of the groups, the modality of treatment varies significantly over time (*p* < 0.0001). Abbreviations: Tx, pre-emptive kidney transplantation; PD, peritoneal dialysis; HD, hemodialysis

The changes in the initial modality of RRT over time are shown in Fig. 2. Hemodialysis was the most common method of initial RRT, with almost three-quarters (62–78%) of patients being initiated on this modality (Fig. 2). The percentage of patients receiving hemodialysis declined slightly over the last decade due to an increase in pre-emptive transplantation and this was higher than in the non-PKD population (Fig. 2). Approximately a quarter of patients (between 24 and 29%) were initiated on peritoneal dialysis and this has remained constant throughout the study periods (Fig. 2).

**Fig. 3:**
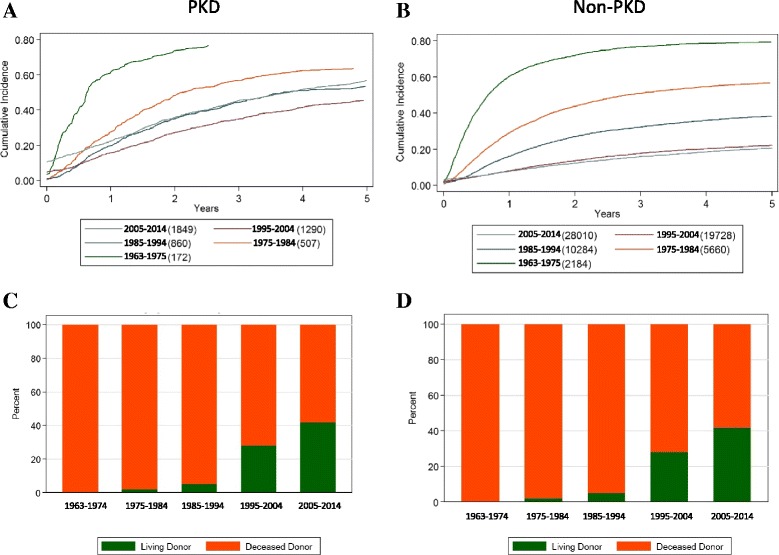
**a** Cumulative incidence of time to first transplant over the five study periods in PKD patients; **b** Cumulative incidence of time to first transplant in non-PKD patients; **c** Proportion of PKD patients receiving a living donor or deceased donor kidney at the time of transplantion over the study period. **d** Proportion of non-PKD patients receiving a living donor or deceased donor kidney at the time of transplantion over the study period

Renal transplant graft survival in PKD patients was similar to non-PKD patients (Table 2). The time to the first renal transplant declined over time, similar to the non-PKD population (Fig. 3a and b). Live donor kidney transplantation has increased since 1990 (Fig. 3c).

**Table 2:** Kidney transplant graft survival in transplanted patients with PKD and other primary kidney diseases (mean percentage and 95% confidence interval [CI])

PKD	0.5 years	1 year	3 years	5 years
1963–1974	58.5 [49.3–0.66.7]	53.7 [44.5–0.62.0]	44.7 [35.8–53.2]	37.4 [28.9–45.9]
1975–1984	56.9 [51.5–61.9]	53.5 [48.1–58.5]	47.1 [41.7–52.2]	40.1 [34.9–45.2]
1985–1994	84.3 [80.9–87.2]	83.2 [79.7–86.1]	78.0 [74.1–81.3]	74.5 [70.5–78.0]
1995–2004	93.0 [90.8–94.7]	91.8 [89.5–93.7]	88.6 [86.0–90.8]	84.2 [81.2–86.8]
2005–2014	97.6 [96.5–98.3]	97.1 [96.0–97.9]	93.9 [92.2–95.2]	90.4 [88.2–92.2]
Non-PKD				
Total				
1963–1974	63.1 [60.7–65.3]	58.1 [55.7–60.5]	48.5 [46.1–0.50.8]	42.1 [39.7–44.4]
1975–1984	63.5 [62.0–65.0]	58.7 [57.1–60.2]	48.4 [46.8–50.0]	42.3 [40.7–43.9]
1985–1994	84.9 [83.9–85.9]	82.7 [81.6–83.7]	74.8 [73.6–76.0]	67.3 [65.9–68.6]
1995–2004	92.7 [92.0–93.3]	91.3 [90.5–92.0]	85.9 [84.9–86.7]	79.7 [78.6–80.7]
2005–2014	95.8 [95.4–96.3]	94.4 [93.9–95.0]	89.1 [88.3–89.8]	83.3 [82.3–84.3]
Diabetic/Vascular				
1963–1974	52.4 [43.3–60.8]	46.8 [37.8–55.2]	36.9 [28.5–45.4]	34.5 [26.2–42.9]
1975–1984	51.9 [46.7–56.8]	46.3 [41.2–51.2]	34.7 [29.9–39.5]	27.8 [23.3–32.3]
1985–1994	83.4 [80.1–86.3]	80.0 [76.5–83.1]	70.8 [66.8–74.3]	61.3 [57.2–65.2]
1995–2004	91.2 [89.1–92.9]	89.5 [87.3–91.4]	82.0 [79.3–84.4]	75.7 [72.6–78.4]
2005–2014	94.7 [93.5–95.7]	93.0 [91.6–94.1]	86.1 [84.1–87.9]	80.1 [77.5–82.3]
Non-vascular				
1963–1974	63.9 [61.5–66.2]	59.0 [56.6–61.4]	49.4 [46.9–51.8]	42.7 [40.3–45.2]
1975–1984	64.8 [63.2–66.4]	60.0 [58.4–61.6]	49.9 [48.3–51.6]	43.9 [42.2–45.6]
1985–1994	85.1 [84.0–86.1]	83.0 [81.8–84.1]	75.4 [74.1–76.6]	68.1 [66.6–69.4]
1995–2004	93.0 [92.2–93.6]	91.6 [90.7–92.3]	86.5 [85.5–87.5]	80.4 [79.3–81.5]
2005–2014	96.1 [95.6–96.6]	94.9 [94.3–95.4]	89.8 [89.0–90.6]	84.1 [83.0–85.2]

*Note*: Non-PKD group shown as “Total” or sub-divided into “Vascular/Diabetes” and “Non-vascular”

### Survival and death rates on renal replacement therapy in PKD

**Fig. 4:**
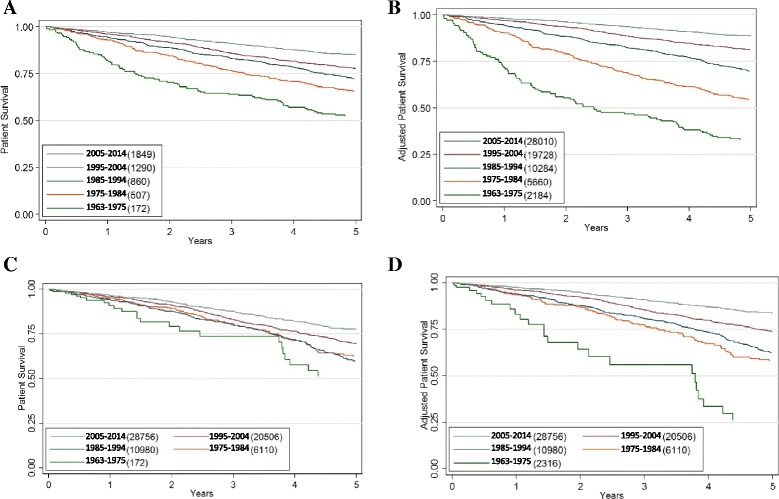
Kaplan-Meier curves showing survival of PKD patients receiving RRT during the study period. Panel **a** shows unadjusted data, whereas Panel **b** shows data adjusted for median age (58 years old). Panel **c** shows unadjusted data (age) but censored at first transplantation, whereas Panel **d** shows adjusted data (age) censored at first transplantation. For all comparisons, the unadjusted and age-adjusted outcomes vary significantly over time (*p* < 0.001)

The unadjusted 5-year survival of ADPKD patients with ESKD improved progressively from 52 to 85% over the study period (Fig. 4a, Table 3). After adjustment for age the 5-year survival improved from 33 to 89% since 1963 (Fig. 4b, Table 4). When censored for kidney transplantation and adjusted for age, the improvement in 5-year survival over the study period was more pronounced, and increased from 26 to 84% (Fig. 4c and d, Tables 5 and 6) and the percentage change between each successive time period was 123, 7, 21, 19 and 7.4% respectively. In all time-periods, survival on RRT in PKD patients was better than in non-PKD patients (Tables 3, 4, 5 and 6).

**Table 3:** Survival rates of incident patients with ESKD (mean percentage and 95% confidence interval [CI])

PKD	0.5 years	1 year	3 years	5 years
1963–1974	90.7 [85.3–94.2]	82.0 [75.4–87.0]	64.0 [56.3–70.6]	52.3 [44.6–59.5]
1975–1984	97.0 [95.1–98.2]	92.9 [90.3–94.8]	76.7 [72.8–80.2]	65.5 [61.2–69.4]
1985–1994	97.0 [95.6–97.9]	94.3 [92.5–95.7]	82.9 [80.2–85.3]	72.1 [69.0–75.0]
1995–2004	98.3 [97.4–98.9]	96.1 [94.9–97.1]	85.8 [83.8–87.6]	77.7 [75.3–79.9]
2005–2014	98.5 [97.8–99.0]	97.1 [96.2–97.8]	91.2 [89.6–92.5]	85.1 [83.1–86.9]
Non-PKD				
Total				
1963–1974	88.1 [86.7–89.4]	79.6 [77.9–81.3]	61.5 [59.5–63.5]	52.4 [50.3–54.5]
1975–1984	93.3 [92.6–93.9]	86.6 [85.7–87.5]	69.9 [68.7–71.1]	58.9 [57.6–60.1]
1985–1994	94.6 [94.2–95.0]	89.2 [88.6–89.8]	68.7 [67.7–69.5]	54.4 [53.5–55.4]
1995–2004	93.8 [93.5–94.1]	87.6 [87.1–88.1]	66.5 [65.9–67.2]	51.1 [50.4–51.8]
2005–2014	94.0 [93.7–94.3]	89.1 [88.7–89.4]	70.4 [69.8–71.0]	55.2 [55.5–56.0]
Diabetic/Vascular				
1963–1974	79.9 [73.4–84.9]	68.8 [61.7–74.9]	43.4 [36.3–50.3]	38.1 [31.2–45.0]
1975–1984	87.0 [84.4–89.1]	76.4 [73.3–79.2]	50.3 [46.8–53.8]	35.6 [32.3–39.0]
1985–1994	92.1 [91.0–93.6]	84.0 [83.6–85.4]	52.6 [50.6–54.4]	33.3 [31.4–35.0]
1995–2004	92.7 [92.2–93.3]	85.1 [84.3–85.8]	58.0 [56.9–59.0]	38.0 [36.9–39.0]
2005–2014	94.0 [93.6–94.3]	88.3 [87.8–88.8]	65.6 [64.7–66.4]	46.6 [45.6–47.6]
Non-vascular				
1963–1974	88.9 [87.5–90.2]	80.7 [78.9–82.3]	63.3 [61.1–65.3]	53.8 [51.6–55.9]
1975–1984	94.3 [93.6–94.9]	88.3 [87.3–89.1]	73.1 [71.8–74.3]	62.6 [61.3–64.0]
1985–1994	95.5 [95.0–95.9]	91.0 [90.3–91.6]	74.2 [73.2–75.2]	61.9 [60.8–63.0]
1995–2004	94.6 [94.2–95.0]	89.5 [88.9–90.0]	72.9 [72.0–73.7]	60.8 [59.9–61.7]
2005–2014	94.0 [93.6–94.4]	90.0 [89.4–90.4]	75.8 [74.9–76.6]	65.7 [63.7–65.7]

*Note*: Non-PKD group shown as “Total” or sub-divided into “Vascular/Diabetes” and “Non-vascular”

**Table 4:** Survival rate (percentage) in incident patients with ESKD (adjusted for median age, 58 years)

PKD	0.5 years	1 year	3 years	5 years
1963–1974	85.0	71.8	46.9	32.8
1975–1984	96.0	90.4	69.0	54.4
1985–1994	97.0	94.3	82.2	69.7
1995–2004	98.7	97.1	88.6	81.1
2005–2014	99.0	98.1	93.7	88.8
Non-PKD				
Total				
1963–1974	78.3	64.1	38.1	27.2
1975–1984	89.7	79.7	55.3	39.9
1985–1994	94.3	88.4	64.8	47.2
1995–2004	95.2	90.1	71.1	54.7
2005–2014	95.5	91.8	76.2	61.7
Diabetic/vascular				
1963–1974	64.5	47.6	18.2	13.7
1975–1984	83.2	70.2	39.8	24.2
1985–1994	92.0	83.6	51.4	30.8
1995–2004	94.1	87.7	63.3	43.0
2005–2014	95.4	91.0	71.9	54.1
Non-vascular				
1963–1974	79.9	65.8	40.3	28.7
1975–1984	91.1	81.7	58.7	43.5
1985–1994	95.1	90.0	70.0	53.7
1995–2004	95.7	91.4	76.1	62.7
2005–2014	95.5	92.3	80.2	69.3

*Note*: Non-PKD group shown as “Total” or sub-divided into “Vascular/Diabetes” and “Non-vascular”

**Table 5:** Survival rate in incident patients with ESKD, censored at transplantation (mean percentage and 95% confidence interval [CI])

PKD	0.5 years	1 year	3 years	5 years
1963–1974	96.2 [90.9–98.4]	90.9 [82.7–95.4]	73.7 [59.3–83.7]	51.3 [34.2–66.0]
1975–1984	98.5 [97.0–99.3]	94.8 [92.2–96.6]	80.8 [75.6–85.0]	62.5 [55.3–68.8]
1985–1994	96.9 [95.5–97.9]	94.0 [92.1–95.5]	79.9 [76.4–82.9]	59.9 [55.0–64.3]
1995–2004	98.3 [97.4–98.9]	95.9 [94.5–96.9]	83.1 [80.5–85.3]	69.4 [66.1–72.5]
2005–2014	98.3 [97.6–98.9]	96.6 [95.5–97.4]	87.6 [85.4–89.5]	77.7 [74.4–80.6]
Non-PKD				
Total				
1963–1974	92.7 [91.4–93.8]	87.1 [85.1–88.8]	66.9 [62.9–70.6]	55.1 [50.1–59.8]
1975–1984	93.8 [93.1–94.4]	87.1 [86.1–88.1]	64.6 [62.9–66.2]	45.9 [43.9–47.9]
1985–1994	94.5 [94.1–95.0]	88.7 [87.9–89.2]	61.9 [60.8–63.0]	40.2 [39.0–41.4]
1995–2004	93.6 [93.3–94.0]	87.0 [86.5–87.5]	62.7 [62.0–63.4]	42.9 [42.1–43.7]
2005–2014	93.7 [93.4–94.0]	88.5 [88.1–88.9]	67.3 [66.6–67.9]	48.5 [47.7–49.3]
Diabetic/Vascular				
1963–1974	83.6 [76.8–88.5]	77.7 [69.7–83.8]	55.6 [42.5–66.8]	51.9 [37.8–64.3]
1975–1984	88.7 [86.1–90.8]	78.1 [74.7–81.0]	47.6 [43.1–52.0]	25.3 [21.0–29.8]
1985–1994	92.0 [90.9–93.0]	83.5 [82.0–84.9]	48.4 [46.4–50.5]	24.9 [23.1–26.8]
1995–2004	92.7 [92.1–0.93.2]	84.8 [84.1–85.6]	56.2 [55.1–57.3]	34.1 [33.0–35.2]
2005–2014	93.9 [93.5–94.3]	88.2 [87.6–88.7]	64.2 [63.3–65.0]	43.5 [42.4–44.5]
Non-vascular				
1963–1974	93.6 [92.2–94.7]	88.0 [86.0–89.8]	68.0 [63.8–71.9]	55.6 [50.3–60.6]
1975–1984	94.6 [93.9–95.3]	88.6 [87.6–89.69]	67.6 [65.8–69.3]	49.8 [47.6–52.0]
1985–1994	95.5 [95.0–95.9]	90.4 [89.7–91.1]	67.7 [66.4–68.9]	47.3 [45.8–48.8]
1995–2004	94.4 [93.9–94.8]	88.7 [88.1–89.3]	68.3 [67.3–69.3]	51.1 [50.0–52.2]
2005–2014	93.5 [93.1–94.0]	89.0 [88.4–89.6]	71.5 [70.5–72.4]	55.7 [54.4–56.9]

*Note*: Non-PKD group shown as “Total” or sub-divided into “Vascular/Diabetes” and “Non-vascular”

**Table 6:** Survival rate (percentage) in incident patients with ESKD, censored at transplantation and adjusted for median age (58 years)

PKD	0.5 years	1 year	3 years	5 years
1963–1974	92.6	83.3	56.0	26.4
1975–1984	98.1	93.4	77.5	58.2
1985–1994	96.9	94.1	80.7	62.5
1995–2004	98.5	96.4	85.5	73.8
2005–2014	98.8	97.5	91.0	83.9
Non-PKD				
Total				
1963–1974	86.1	76.4	47.4	33.7
1975–1984	91.2	82.4	56.1	37.1
1985–1994	94.1	87.7	60.8	39.0
1995–2004	94.4	88.6	66.6	47.5
2005–2014	95.0	90.8	73.0	56.0
Diabetic/vascular				
1963–1974	69.1	59.3	29.2	25.2
1975–1984	86.7	74.7	42.4	20.6
1985–1994	91.9	83.3	48.2	24.8
1995–2004	93.6	86.7	60.4	38.5
2005–2014	95.1	90.4	69.9	50.7
Non-vascular				
1963–1974	87.8	78.1	49.4	34.8
1975–1984	92.2	83.9	58.9	40.6
1985–1994	94.9	89.5	66.4	45.9
1995–2004	95.0	90.0	71.7	55.3
2005–2014	94.8	91.1	76.7	62.8

**Fig. 5:**
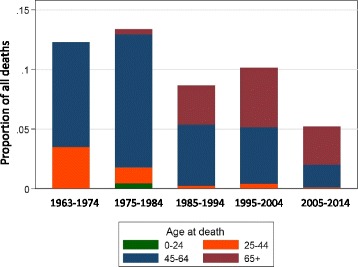
The proportion of deaths due to cerebrovascular accident over the study period

**Fig. 6:**
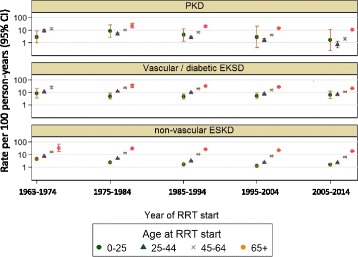
Mortality rates (per 100 person-years) during RRT by age and year of RRT start and category of primary renal disease. The variation in rates among time periods, age categories and disease categories are all significant (*p* < 0.001) and mutually independent

There were a total of 2013 deaths during the study period with an overall death rate of 6.2 deaths per person year. The death rate was lowest in the 25–44 year age group and highest in the >65 year old group [<25 years: 4.4 (1.6–11.7); 25–44 years: 2.7 (2.2–3.4); 45–64 years: 4.4 (4.1–4.6); >65 years: 11.9 (11.2–12.6)]. Death due to cerebrovascular accidents were a small proportion of the overall cause of mortality, and declined from 16% in 1970–1979 to 6% during between years 2000 and 2011 (Fig. 5). The death rate, as expressed according to age category, has progressively declined in PKD group (Fig. 6). However, the improvement in death is less marked in the non-PKD groups (Fig. 6).

## Discussion

The development of end-stage kidney disease is the most significant clinical complication of PKD. In this study we investigated the incidence rate, age of onset and survival of PKD patients receiving RRT in Australia and New Zealand over a 50-year period to evaluate whether time-dependent changes have occurred. To our knowledge, this is the longest longitudinal follow-up of ESKD patients with PKD. The main findings were that: (i) the median age of onset and incidence of RRT has increased but relatively stable since 1990; (ii) the proportion of patients older than 65 years starting RRT has increased since 1990, representing almost a quarter of the cohort; (iii) hemodialysis was the most common initial dialysis RRT modality and technique survival on peritoneal dialysis was not affected by PKD; (iv) the percentage of pre-emptive kidney transplantation as the initial RRT modality has increased; and (v) the 5-year survival of PKD patients on RRT has continually improved over time.

Previous studies indicate that the population prevalence of PKD ranges anywhere from 1 in 400 to 1 in 5000 [1, 18–23]. This wide variation is probably because the true population prevalence of PKD is virtually impossible to determine accurately, as disease penetrance (diagnosed by renal ultrasound) peaks late in life (after the age of 60 years), and a high frequency of patients (up to 50% or more) have a relatively benign life-time course and do not come to medical detection and/or the develop ESKD [20]. On the other hand, the increasing detection of asymptomatic PKD due to the fact that more diagnostic tests are being performed for other reasons in developed nations, may falsely give the impression that the population-based prevalence of PKD is rising [13]. Therefore, dialysis registry data in many ways provides the most accurate method of capturing changes in the pattern of disease incidence in the subset of PKD patients at high risk of renal morbidity.

The results of the current study show that that the incidence of ESKD due to PKD has progressively increased over the past five decades. Cohort studies from Denmark and the United States have made the same observation [9, 11], and these common findings are likely to be a reflection of a global increase in the age of patients starting dialysis rather than a feature specific to PKD. For instance, the median age at starting dialysis patients in Australia and New Zealand was 59.5 years in 1998 whereas it had increased to 62.9 years for Australia and to 60.3 years for New Zealand in 2009 [17]. This rising incidence is therefore common to all types of chronic kidney disease, including increased access and availability of RRT, and increased propensity to offer RRT to older patients, as well as reductions in the competing risk of cardiovascular mortality among those with pre-dialysis chronic kidney disease [24, 25].

Recent clinical trials in PKD suggest that a lower blood pressure and the use of renin-angiotensin inhibitors reduces the growth rate of renal cysts in PKD (albeit to a small extent of ~14% over an 8 year period) but do not alter the long-term decline in renal function [15]. In the current study the median age of onset of ESKD increased in PKD patients over time but has been stable since 1990. Angiotensin-converting enzyme inhibitors were introduced in Australia in the early 1980s (captopril in 1983 and enalapril in 1986), and first reported to have renoprotective properties in diabetic kidney disease in 1992 [26]. However, the real-world utilisation of renin-angiotensin inhibitors in CKD patients varies between 58 and 64% [27–29], and hence a longer period of registry follow-up is required to evaluate their impact on the incidence of ESKD in ADPKD. Low birth weight has been also linked to an earlier onset of ESKD but has not been specifically addressed in the current study [30]. Therefore, the increasing age of onset of ESKD prior to 1990 is more likely due to factors discussed earlier (that is, improved access to RRT and reductions in cardiovascular death prior to RRT) [13]. Assessment of renal function by eGFR was incrementally introduced 2005 onwards [31] but it is too premature to determine the impact (if any) this could have on the incidence of ESKD. In this regard, one caveat to these conclusions is that the rate of renal disease progression in PKD occurs slowly over many decades [32]. For example, a recent analysis suggested that the time taken to transition from the early stage chronic kidney disease to final phase of ESKD, is up to seven times slower in PKD than in that due to other causes (e.g., 17.1 years in PKD vs. 2.5 years in non-PKD diseases) [33]. Thus, there may be a significant lag-time for subtle improvements in pre-dialysis medical management to be discerned, even with long-term registry data. Additionally, in this study it is not possible to model potential factors that may influence the changes in incidence using registry data because relevant risk factors (such as the use of ACE inhibitors) are not part of routine ANZDATA data collection. Instead, in the present study we have analyzed temporal changes in the incidence of the EKSD due to PKD and then associated them with changes in therapeutics in the Australia and New Zealand. Future and ongoing prospective studies, such as the CRISP cohort [34], are more likely to elucidate mechanisms for the underlying changes in incidence.

Hemodialysis was the most common mode of initial RRT in PKD patients in Australia and New Zealand, with only 25% using peritoneal dialysis. This pattern of dialysis modality utilization remained stable throughout the study period. It is not clear whether the preference for hemodialysis was due to patient-dependent and/or health-care-related biases. Certainly, it has been hypothesized that PKD patients experience greater technique failure on peritoneal dialysis due to systemic complications such as hernias, diverticular disease and reduced peritoneal surface area because of nephromegaly [35–37]. However, this hypothesis was not been supported by others [38]. Lobbedez et al. [39] analyzed 344 French PKD and did not find evidence of a higher incidence of technique failure when compared with the non-PKD group. Similarly, Portoles et al. [40] also found that a “peritoneal dialysis first” model combined with an active transplant program has been successful in Spain. Li et al. [41] also reported peritoneal dialysis is a feasible treatment option for PKD patients despite a higher risk of abdominal wall hernia in their analysis of 126 peritoneal dialysis patients from Hong Kong. Finally, Courivad et al. also recently verified that the total kidney volume in PKD patients did not predict technique survival on peritoneal dialysis [42].

The survival of PKD patients in Australia and New Zealand improved with each decade, similar to other cohorts [43]. In our study, the magnitude of increase in the 1-year survival was most marked between the first two time periods (1963–1974 to 1975–1984). The progressive increase in survival could, in part, be explained by the rise in living kidney transplantation in the study cohort. The current data are similar to that of Orskov et al. [11] who followed 693 Danish PKD dialysis patients for 18 years (1990–2007) and found that there was a progressive improvement in survival (2-year survival increased from 80 to 86%, and 5-year survival increased from 56 to 68%). It is noteworthy that cerebrovascular disease as a cause of death has decreased in PKD dialysis patients in our study period. Presumably this is a result of increased radiological screening for cerebral aneurysms over the study period and improvements in the management of hypertension [44]. These data are also consistent with the Danish cohort of PKD dialysis patients, where it was reported that cerebrovascular declined by 69% over an 18-year period [45].

There are several limitations of our study. First, it is a retrospective registry-based analysis and therefore provides only correlative data associated with the timing of changes in medical treatments, such as the introduction of angiotensin converting enzyme inhibitors in Australia. Second, the categorization of primary renal disease used by the ANZDATA Registry has remained constant for many years. We hypothesize the vast majority of patients in the cohort are likely to have ADPKD because this is by the most common form of cystic renal disease associated with CKD and ESKD in adults, with an estimated population prevalence of ~1:2500 (as discussed earlier in the discussion) whereas the autosomal recessive variant falls into the ultra-rare category with an estimated incidence of 1:20,000 to 40,000 [46] and almost all manifest at birth or early childhood. In this regard, the latter are more likely to have been reported under the “juvenile polycystic disease” category, not included in our analysis. At present, ANZDATA does not collect information regarding genetic testing because this is not routine clinical practice in Australia (and most other countries), where the Pei-Ravine Unified Ultrasound Criteria is the standard method of diagnosis [47]. The latter has high sensitivity and specificity (validated against DNA Sanger Sequencing), and we would anticipate that the majority of cases identified as PKD in the ANZDATA Registry would be of the autosomal dominant subtype. Third, as discussed earlier, it may take a longer period of follow-up before minor changes in the incidence of ESKD due to PKD are consistently detected as a result of changes in pre-dialysis care. Finally, another limitation is the year in which the entire (100%) Australian and New Zealand population had full access to dialysis. Although an exact date cannot be provided, on reviewing the rate of expansion of the entire ESKD population (data not shown), we noted that the absolute number of patients was more stable (that is, the rate slowed) from 1970 onwards, and therefore hypothesise that this period onwards was period in which the entire population would have had full access to dialysis treatment options.

## Conclusions

In conclusion, the results of this study show that the incidence and age of onset of ESKD in patients with PKD has increased over a 50-year period in Australia and New Zealand but have stabilized in the most recent era. These findings are important because they clearly imply that that the development of disease-specific drugs that alter renal cyst growth [48] are needed to prevent kidney failure due to PKD. Because renal cysts in PKD develop during early childhood, it does not seem unreasonable to suggest that the implementation of highly effective and safe disease-specific treatments in a young person with PKD [49] could potentially eliminate the development of ESKD due to this condition in the 21^st^ century. Further studies will hopefully confirm this hypothesis in the future.

## Abbreviations

ADPKD: Autosomal dominant polycystic kidney disease
ANZDATA: Australia and New Zealand Dialysis and Transplant Registry
ESKD: End-stage kidney disease
PKD: Polycystic kidney disease
RRT: Renal replacement therapy
